# Treatment strategies in patients with diabetes and three‐vessel coronary disease: What should we choose?

**DOI:** 10.1186/s12933-021-01241-6

**Published:** 2021-02-11

**Authors:** Bo Liang, Ning Gu

**Affiliations:** 1 Nanjing University of Chinese Medicine, Nanjing, China; 2 Nanjing Hospital of Chinese Medicine Affiliated to Nanjing University of Chinese Medicine, Nanjing, China

## Abstract

The recent study demonstrating that percutaneous coronary intervention and coronary artery bypass grafting were associated with a lower risk of death and major adverse cardiac and cerebrovascular events (composite of all-cause death, myocardial infarction, or stroke) than with medical therapy among patients with diabetes and triple-vessel disease was very interesting. However, the nature of single-center nonrandomized and nonblinded studies that are not placebo controlled limits the extrapolation and generalizability of the results. As a result, the existing body of evidence does not fully support the use of revascularization treatment strategies in patients with diabetes and triple-vessel disease. Importantly, the safety of revascularization treatment strategies in this particular population remains uncertain. Therefore, further studies are needed to assess the risks and benefits of comprehensive treatment in these patients.

As an independent risk factor for cardiovascular disease, diabetes greatly increases the incidence and mortality of cardiovascular disease [1–3]. Simultaneously, patients with diabetes are at increased risk of having a cardiovascular event and are more likely to have diffuse and multivessel vascular lesions [4, 5], making coronary revascularization challenging [6]. Such patients are prone to a more rapid progression of atherosclerosis, significantly increasing the need for myocardial revascularization.

The present Commentary refers to the recently published article by Zhao et al. [7] describing that, in patients with diabetes and triple-vessel disease (TVD), percutaneous coronary intervention (PCI) and coronary artery bypass grafting (CABG) were associated with a lower risk of death and major adverse cardiac and cerebrovascular events (MACCE, composite of all-cause death, myocardial infarction, or stroke) than medical therapy (MT). The authors conducted a prospective and single-center study that included 3117 patients with diabetes and TVD (defined as angiographic stenosis of ≥ 50% in all three main epicardial coronary arteries, with or without left main artery involvement) from Fuwai Hospital (Beijing, China) with a median follow-up of 6.3 years to explore whether MT or surgical treatment strategies (PCI and CABG) are the most beneficial in this special population. First, it should be noted that at baseline, before all patients received the corresponding treatment, some susceptibility factors (age, sex, peripheral arterial disease history, CABG history, revascularization history, creatinine clearance, left ventricular ejection fraction, and synergy between percutaneous coronary intervention with taxus and cardiac surgery (SYNTAX) score) were not balanced between the patients in the different groups. We all know that in real-world studies, especially single-center studies, baseline inequality is a common thing [8]; accordingly, we need to be careful when extending the conclusions to all populations. We are looking forward to a multicenter, prospective, randomized controlled trial to verify these findings. Second, fortunately, the authors performed a detailed subgroup analysis for different SYNTAX scores, and the results showed that when all-cause death was the clinical endpoint, regardless of the SYNTAX subgroups, patients in the PCI and CABG groups showed a significantly lower risk than those in the MT group. However, when MACCE was the clinical endpoint, PCI failed to reduce the risk of MACCE compared with MT (*P* = 0.559) in patients with a SYNTAX score of ≤ 22. Third, in this population, it may be difficult to choose the optimal revascularization strategy. The outcomes of CABG or PCI have been extensively evaluated. The authors found that the risk of death in the PCI group (HR 0.40, 95% CI  0.32 ~ 0.51, *P* < 0.0001) and the CABG group (HR 0.33, 95% CI 0.26 ~ 0.44, *P* < 0.0001) was significantly lower than that in the MT group, while the CABG group had a lower risk of death compared with the PCI group with no significant difference (HR 0.83, 95% CI 0.62 ~ 1.18, *P* = 0.2215); the risk of MACCE in the PCI group (HR 0.71, 95% CI 0.60 ~ 0.84, *P* < 0.0001) and the CABG group (HR 0.48, 95% CI 0.39 ~ 0.57, *P* < 0.0001) was significantly lower than that in the MT group, while the CABG group had a lower risk of MACCE compared with the PCI group (HR 0.67, 95% CI 0.55 ~ 0.81, *P* < 0.0001), which are not consistent with our conclusions that CABG group has lower all-cause mortality, cardiac mortality, recurrent myocardial infarction, and repeat revascularization and higher cerebrovascular accident than the PCI group by integrating 6 randomized controlled trials (ARTS, BARI 2D, FERRDOM/FERRDOM Follow-On, MASS II, SYNTAX, and VACARDS). Finally, the authors focus on the effectiveness but ignore the safety. Safety is also a very important factor to consider when clinicians make decisions.

In summary, the results presented in the existing body of evidence do not fully support the use of revascularization treatment strategies in patients with diabetes and TVD. Importantly, the safety of revascularization treatment strategies in this particular population remains uncertain. Therefore, further studies are needed to assess the risks and benefits of comprehensive treatment in those patients.

## Data Availability

Not applicable.

## Abbreviations

CABG: Coronary artery bypass grafting
MACCE: Major adverse cardiac and cerebrovascular events
MT: Medical therapy
PCI: Percutaneous coronary intervention
SYNTAX: Synergy between percutaneous coronary intervention with taxus and cardiac surgery
TVD: Triple-vessel disease
